# The complete genome sequences of *Bacillus velezensis* B26: a promising biocontrol agent and biofertilizer

**DOI:** 10.12688/f1000research.160546.1

**Published:** 2025-02-06

**Authors:** Venkatesh Kamath B, Sandeep Mallya, Subrahmanyam Volety Mallikarjuna, Kiran Kumar Kolathur

**Affiliations:** 1 Department of Pharmaceutical Biotechnology, Manipal College of Pharmaceutical Sciences (MCOPS), Manipal Academy of Higher Education, Manipal, Karnataka, 576104, India; 2 Manipal School of Life Sciences, Manipal Academy of Higher Education, Manipal, Karnataka, 576104, India

**Keywords:** Genome sequencing, Biocontrol agent, Plant growth promotion, Pangenome analysis

## Abstract

*Bacillus velezensis* is a bacterium widely recognized for its biocontrol properties and ability to promote plant growth. This study presents the whole-genome sequence of *B. velezensis* B26, a newly identified strain isolated from chicken carcass soil in Udupi, India. The bacterium showed strong activity against fungal pathogens and exhibited diverse enzymatic activities. The whole-genome sequencing was executed using Illumina technologies. Assembly revealed that strain B26 possesses a genome of 3,946,698-bp with a G+C content of 46.3%. Genome annotation identified 3776 protein-coding genes, 1 rRNA gene, 50 tRNA genes, 5 ncRNA genes, and 59 pseudogenes. Functional analysis of the *B. velezensis* B26 genome revealed 216 genes involved in carbohydrate metabolism, 3 genes in potassium metabolism, 148 genes linked for cofactors, vitamins, prosthetic groups and pigments, 10 genes involved in phosphorus metabolism, 24 genes associated with iron acquisition and metabolism, 20 genes for nitrogen metabolism, 6 genes involved in sulfur metabolism, 6 genes in secondary metabolism, 12 genes associated with metabolism of aromatic compounds, 43 genes involved in stress response and 36 genes associated with virulence, disease and defence. The raw sequence data generated in this work have been deposited in the NCBI database and the genome sequence is available under the accession number JAYKOV000000000. These genomic data provide insight into the biocontrol ability and plant-growth promoting capabilities of *B. velezensis* B26.

## Introduction

*Bacillus velezensis* is a valuable bacterium that is extensively used for several biotechnological applications to promote plant growth. For instance, the *B. velezensis* LT1 strain displayed antifungal activity against the soil-borne fungal plant pathogen *Sclerotium rolfsii* LC1. *B. velezensis* LT1 secretes a diverse antimicrobial compound that inhibit the growth of *S. rolfsii* LC1 ( Tang et al. 2024). Likewise, two *B. velezensis* strains 5YN8 and DSN012 act as biocontrol agents against *Botrytis cinerea*, a pathogen that causes severe gray mold disease in crops. These strains inhibit spore formation and the growth of *B. cinerea* through the production of certain secondary metabolites or volatile organic compounds ( Jiang et al. 2018). Several strains of *B. velezensis* isolated from different sources produce diverse secondary metabolites to control plant pathogens. For example, *B. velezensis* HNA3 produced several secondary metabolites. Similarly, several antimicrobial metabolites to control plant pathogens were produced by different *B. velezensis* strains ( Zaid et al. 2022; Rabbee et al. 2023). Besides anti-microbial activity, another *B. velezensis* SQR9 strain implicated in promoting plant growth by enhancing root colonization through biofilm formation ( Xu et al. 2019). Another strain, *B. velezensis* Ag75, has been identified as a biocontrol agent, phosphate solubilizer and biofertilizer for maize and soybean crops ( Mosela et al. 2022).

The plant growth promoting and biocontrol capabilities of several *B. velezensis* strains were deciphered using the genomic sequencing and analysis. These studies were key to understand organism’s capability to produce antimicrobial compounds, enzyme secretion or secondary metabolites that promotes plant growth. For example, whole genome sequencing of *B. velezensis* HNA3 led to establish several genes clusters associated in promoting plant growth. Among these genes, major percentage of genes are involved in amino acid metabolism, carbohydrate transport, and secondary metabolite biosynthesis (S. et al. 2022). Similarly, the *B. velezensis* CH1 strain was isolated from high-quality oats, exhibited antimicrobial properties contributing to oat growth and resistance to infections. Comparative analysis of the CH1 strain revealed 13 gene clusters linked with production of 15 secondary metabolites with antimicrobial properties. Furthermore, the strain harboured numerous putative genes for indole acetic acid (IAA) production, spermidine and polyamine synthesis. These results indicate that the possible applications of *B. velezensis* CH1 as a biofertilizer ( Cheng et al. 2024).

In this study, we report genome sequences of *B. velezensis* B26, which was isolated from soil samples collected from a chicken carcass, Manipal, Udupi, India (Location: 13.325922, 74.804554). In our screening assay to identify chondroitinase producers, *B. velezensis* B26 was identified. This strain demonstrated potent antifungal capabilities against the opportunistic fungal pathogen *Candida albicans* and the emerging fungal pathogen *Saccharomyces cerevisiae* ( http://doi.org/10.7324/JAPS.2025.200474). This study aims to provide a comprehensive genomic analysis of *B. velezensis* B26, with a focus on identifying genetic traits that contribute to its biotechnological potential.

## Methods

### Whole genome analysis of bacteria

The genome of *B. velezensis* B26 strain was analysed to explore its microbial genomic characteristics. HiMedia Laboratories Pvt Ltd, Maharashtra, INDIA performed the whole-genome analysis.

### Sample preparation workflow

Genomic DNA (gDNA) from *B. velezensis* B26 was isolated using DNA Mini Kit (Qiagen). The integrity of the gDNA was assessed spectrophotometrically to measure the A260/280 ratio and its concentration. For library preparation, 250ng of DNA was processed with the QIASeq FX DNA Kit (Qiagen), following manufacturer’s protocol to generate fragmented, adapter-ligated and indexed libraries. The Illumina NextSeq 550 with 300-cycle paired-end sequencing chemistry was employed for sequencing the finalized library.

### Tapestation analysis of NGS libraries

For fragment analysis, 2 μL of high-sensitivity D1000 sample buffer and 2 μL of the final library sample were mixed using vortexer for 1 min. The mixture was then loaded onto the D1000 ScreenTape and analyzed using the Agilent 4200 TapeStation System. This system employs DNA electrophoresis to separate fragments up to 1000 base pairs. The trace analysis revealed an average fragment size of 301 base pairs, which, along with the library concentration, indicates the library’s suitability for next-generation sequencing.

### Analysis workflow

In data analysis all reads are checked for quality and then the quality control (QC’)-quality reads are passed through the process flows simultaneously. The detailed steps involved in each process flow are included within each section below.

### Reads quality control

Briefly, raw fastq files were verified using FastQc v0.11.9.8 The details of parameters checked are added in a MultiQC report ( Ewels et al. 2016) (Supplementary file 1). The fastp tool (v0.12.4) was utilized to remove adapter contamination from the sequencing data ( Chen et al. 2018). A quality score of Q30 or higher indicates a basecall accuracy of 99.9%, meaning that only 1 in 1000 bases is likely to be incorrect. The resulting files for each read were subjected for genome assembly.

### Genome assembly and annotations

A denovo assembly using a De-Brujin graph was performed to organize the short DNA reads into longer contiguous sequences, referred to as contigs. These contigs served as a starting material used for performing a genome annotation, facilitating the assignment of functional roles to various genomic regions of the organism under investigation.

The quality of the genome assemblies produced by the Spades ( Bankevich et al. 2012) and Megahit ( Li et al. 2015) assemblers was evaluated using the Quast tool ( Gurevich et al. 2013). Compared with the megahit assembler, the Spades assembler had longer assembled contigs with better N50 values. Hence, the assembly generated by Spades was selected for further downstream analyses.

### RAST Server: Rapid annotations using subsystems technology

The RAST server is an automated platform developed for the bacterial genome annotation. It detects protein-coding regions, along with genes coding for ribosomal and transfer RNAs, allots functional roles to these elements ( Aziz et al. 2008). Additionally, it allows comparative analysis through the SEED environment ( Overbeek et al. 2014). The RAST server remains a viable tool for efficient and reliable genome annotation.

## Results

### Whole-genome analysis of *B. velezensis* B26

**Figure 1.**
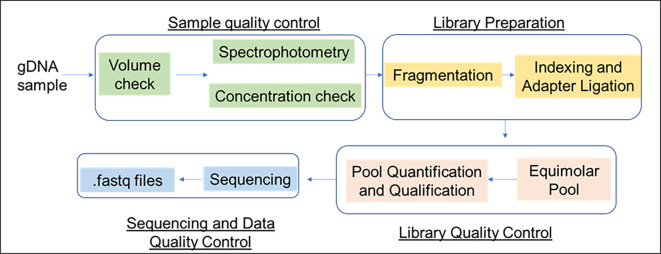
Sample preparation and sequencing workflow.

We performed a genome analysis of *B. velezensis* B26 to understand the biochemical potential of the organism. Figure. 1 depicts the sample preparation and sequencing workflow. Importantly, the assembled genomes may not be 100% complete. However, the assembly generated here meets the standards acceptable to general bioinformatics repositories as part of the data gathered for a publication. These whole-genome shotgun data have been provided with the accession number JAYKOV000000000 (NCBI database). The master record data is available from our various Entrez servers. The individual sequences are available from a hyperlink at the bottom of the WGS master record JAYKOV000000000.

The genome assembly method employed was Megahit v. 2023-07-12 tool, the genome representation was full, the genome coverage was 100.0x and the sequencing technology employed was Illumina. Genome annotation was carried out using the NCBI Prokaryotic Genome Annotation Pipeline (PGAP) ( Haft et al. 2018; Li et al. 2021). The annotation results are summarized in Table 1, which provides an overview of the genome features and information. Table 2 summarizes key assembly statistics, including the number of contigs, total length of the sequenced genome, and the number of proteins annotated.

**Table 1.** Genome annotation data for *Bacillus velezensis* B26.

##Genome-Annotation-Data-START##	
Annotation Provider:	NCBI
Annotation Date:	01/04/2024 08:32:37
Annotation Pipeline:	NCBI Prokaryotic Genome Annotation Pipeline (PGAP)
Annotation Method:	Best-placed reference protein set; GeneMarkS-2+
Annotation Software revision:	6.6
Features Annotated:	Gene; CDS; rRNA; tRNA; ncRNA
Genes (total):	3,891
CDSs (total):	3,835
Genes (coding):	3,776
CDSs (with protein):	3,776
Genes (RNA):	56
rRNAs:	1 (5S)
complete rRNAs:	1 (5S)
tRNAs:	50
ncRNAs:	5
Pseudo Genes (total):	59
CDSs (without protein):	59
Pseudo Genes (ambiguous residues):	0 of 59
Pseudo Genes (frameshifted):	40 of 59
Pseudo Genes (incomplete):	32 of 59
Pseudo Genes (internal stop):	8 of 59
Pseudo Genes (multiple problems):	19 of 59
##Genome-Annotation-Data-END##	
##Genome-Assembly-Data-START##	
Assembly Method:	Megahit v. 2023-07-12
Genome Representation:	Full
Expected Final Version:	Yes
Genome Coverage:	100.0x

**Table 2.** Assembly statistics for *B. velezensis* B26.

# of Contigs:	31
# of Proteins:	3,776
Total length:	3,947,698 bp
BioProject:	PRJNA1060932
BioSample:	SAMN39250459
Keywords:	WGS
Annotation:	Contigs
Organism:	Bacillus velezensis – show lineage
Biosource:	/collected_by = Manipal College of Pharmaceutical Sciences (MCOPS) /collection_date = 2019-12-01 /country = India: Udupi /isolation_source = environmental /mol_type = genomic /strain = B26
WGS:	JAYKOV010000001:JAYKOV010000031
Reference:	Evaluation of bioactivities of the bacterial strain Bacillus velezensis B26: Unpublished
Submission:	Submitted (04-JAN-2024) Pharmaceutical Biotechnology, Manipal College of Pharmaceutical Sciences (MCOPS), MAHE, Madhav Nagar, Manipal, Karnataka 576104, India – Kolathur,K.K.

### Identification of subsystem features by The RAST Server: Rapid Annotations using Subsystems Technology (RAST)

**Figure 2.**
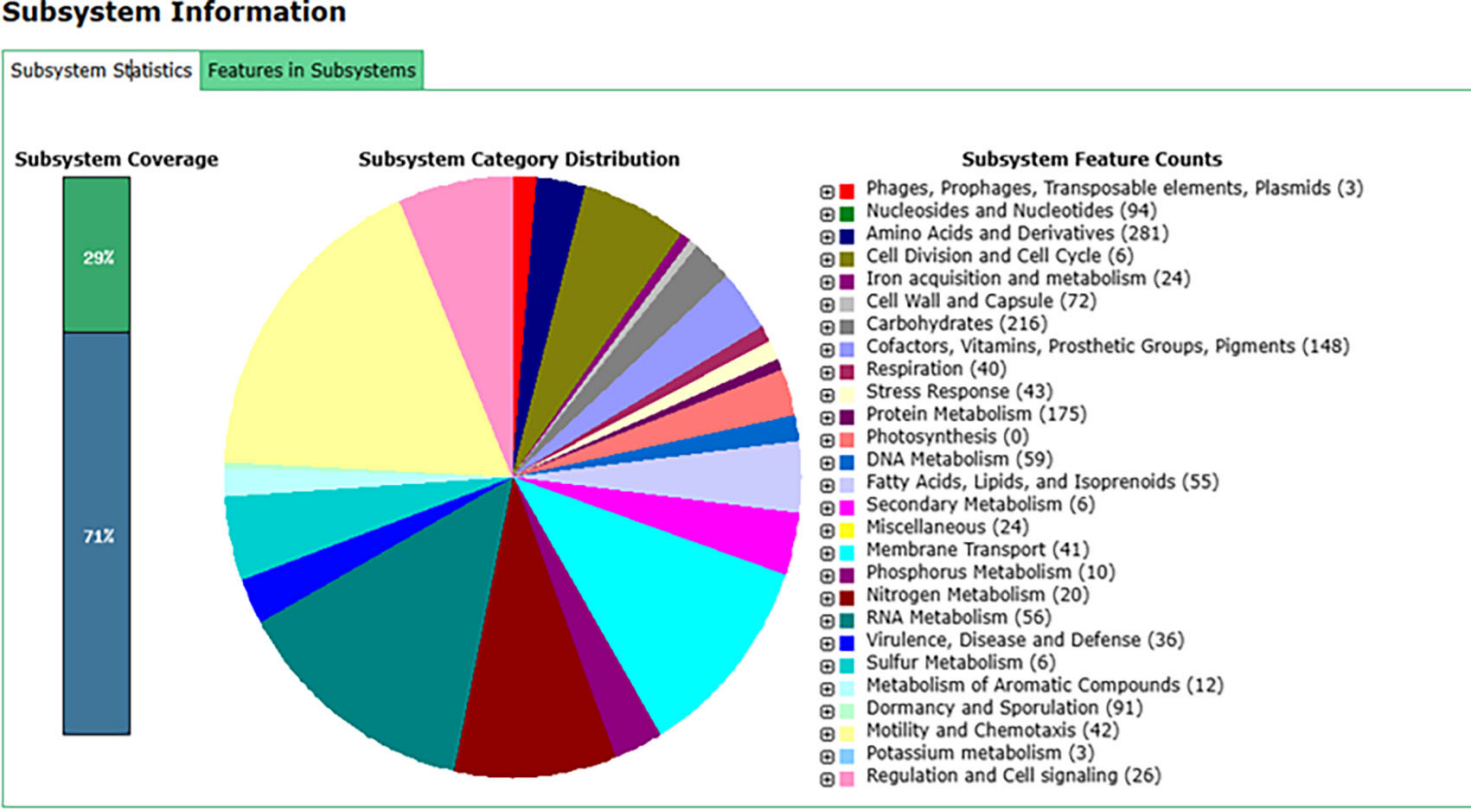
Subsystem statistics of the *B. velezensis* B26 genome annotations. The genome of *B. velezensis* B26 was annotated using the RAST server, and the features in subsystem are compared within the SEED environment. The figure highlights key subsystems identified in the genome, including genes associated with carbohydrate metabolism, stress response, virulence, disease and defense, as well as genes involved in metabolism.

In addition to utilizing the NCBI PGAP, we also submitted the sequenced genome data of *B. velezensis* B26 to the RAST server. This service provides fully automated annotation for bacterial genomes. Using the RAST annotation platform, we recognized genes coding for proteins, ribosomal and transfer RNAs, assigned functional roles to these genes, and predicted the subsystems present in the *B. velezensis* B26 genome (Supplementary Table 1). Furthermore, the annotated genome is presented on a platform that allows for comparative analysis in the SEED environment ( Figure 2).

Our analysis revealed the presence of 216 genes involved in carbohydrate metabolism, 3 genes related to phages, prophages, transposable elements, and plasmids, 43 genes for stress response, and 35 genes linked to virulence, disease and defense. The genes related to the stress response and virulence might be responsible for the biocontrol properties of *B. velezensis* B26 (Supplementary Table 2).

Additionally, several genes involved in metabolic processes were identified, including 59 genes related to DNA metabolism, 10 related to phosphorus metabolism, 20 related to nitrogen metabolism, 6 related to sulfur metabolism, 12 related to metabolism of aromatic compounds, 3 related to potassium metabolism, 10 related to phosphorus metabolism, and 24 related to acquisition and metabolism. These metabolic pathways suggest potential biofertilizer activity (Supplementary Table 2).

### *B. velezensis* B26 pangenome analysis

Pangenome analysis provides valuable understandings into the genome of prokaryotes. Using Roary software, a large-scale pangenomes are constructed by identifying both core and accessory genes. This method aids in understanding the conserved genes within an organism, as well as accessory genome, to understand the fundamentals linked with natural selection and evolutionary dynamics.

Pangenome analysis of *B. velezensis* B26 identified a total of 5,576 genes. Among these genes, 3,480 genes were considered core genes and were found in 99% to 100% of strains. A total of 1, 143 genes were classified as shell genes, which were present in 15% to 95% of strains, whereas 953 genes were identified as cloud genes, which were found in fewer than 15% of strains ( Table 3). These analyses indicate that the major portion of the genome is conserved across species.

**Table 3.** Pangenome analysis of *B. velezensis* B26.

	Gene_type	Criteria	Number_of_genes
1	Core genes	(99% <= strains <= 100%)	3480
2	Soft core genes	(95% <= strains < 99%)	0
3	Shell genes	(15% <= strains < 95%)	1143
4	Cloud genes	(0% <= strains < 15%)	953
5	Total genes	(0% <= strains <= 100%)	5576

## Conclusion

The whole-genome analysis, genome annotation, and pangenome analysis of *B. velezensis* B26 deciphered the key genes responsible for its biotechnological applications. A huge number of protein coding genes (3776) responsible for several cellular functions were identified. Importantly, presence of gene cluster coding for stress response, virulence, and defense indicate that *B. velezensis* B26 can effectively inhibit plant pathogens. Further, presences of genes associated with nitrogen, phosphorus, and sulfur metabolism might suggest the strain’s ability to act as a biofertilizer, supporting plant growth. Altogether, these findings can pave the way for further exploration of *B. velezensis* B26 in agricultural applications.

## Ethics and consent

Ethical approval and consent were not required.

## Data Availability

NCBI Nucleotide Database: Whole-genome sequencing data of Bacillus velezensis B26. Accession number JAYKOV000000000; https://www.ncbi.nlm.nih.gov/nuccore/JAYKOV000000000. Contigs view; https://www.ncbi.nlm.nih.gov/Traces/wgs/JAYKOV01?display=contigs. Whole-genome shotgun project; https://www.ncbi.nlm.nih.gov/Traces/wgs/JAYKOV01. These data support the findings of this study and are publicly accessible. The complete genome sequence and associated supplementary data for *Bacillus velezensis* B26 are available on **Figshare**. **Figshare**: Supplementary file 1 for the complete genome sequences of *Bacillus velezensis* B26 study. DOI: 10.6084/m9.figshare.28107950. The project contains the following underlying data:
•
**Supplementary Table 1**: Complete genome sequences of *Bacillus velezensis* B26. DOI: 10.6084/m9.figshare.28107941. •
**Supplementary Table 2**: Complete genome sequence for *Bacillus velezensis* B26. DOI: 10.6084/m9.figshare.28107947. Data are available under the terms of the Creative Commons Attribution 4.0 International license (CC-BY 4.0).
