# The complete genome sequences of *Bacillus velezensis* B26: a promising biocontrol agent and biofertilizer

**DOI:** 10.12688/f1000research.160546.2

**Published:** 2025-07-08

**Authors:** Venkatesh Kamath B, Sandeep Mallya, Subrahmanyam Volety Mallikarjuna, Kiran Kumar Kolathur

**Affiliations:** 1 Department of Pharmaceutical Biotechnology, Manipal College of Pharmaceutical Sciences (MCOPS), Manipal Academy of Higher Education, Manipal, Karnataka, 576104, India; 2 Manipal School of Life Sciences, Manipal Academy of Higher Education, Manipal, Karnataka, 576104, India

**Keywords:** Genome sequencing, Biocontrol agent, Plant growth promotion, Pangenome analysis

## Abstract

*Bacillus velezensis*, is a bacterium widely recognized for its biocontrol properties and ability to promote plant growth. This study presents the whole genome sequence of *B. velezensis* B26, a newly identified strain isolated from chicken carcass soil, Udupi, India. The bacterium showed strong activity against fungal pathogens and exhibited diverse enzymatic activities. The whole genome sequencing was executed using Illumina technologies. Assembly revealed that strain B26 possesses a genome of 3,946,698-bp with a G+C content of 46.3%. Genome annotation identified 3776 protein-coding genes, 1 rRNA gene, 50 tRNA genes, 5 ncRNA genes, and 59 pseudogenes. Functional analysis of the *B. velezensis* B26 genome revealed 216 genes involved in carbohydrate metabolism, 3 genes in potassium metabolism, 148 genes linked for cofactors, vitamins, prosthetic groups and pigments, 10 genes involved in phosphorus metabolism, 24 genes associated with iron acquisition and metabolism, 20 genes for nitrogen metabolism, 6 genes involved in sulfur metabolism, 6 genes in secondary metabolism, 12 genes associated with metabolism of aromatic compounds, 43 genes involved in stress response and 36 genes associated with virulence, disease and defense. The raw sequence data generated in this work has been deposited in the NCBI database and the genome sequence is available under the accession number JAYKOV000000000. This genomic data provides insight into the biocontrol ability and plant-growth promoting capabilities of *B. velezensis* B26.

## Introduction

*Bacillus velezensis* is a valuable bacterium that is extensively used for several biotechnological applications to promote plant growth. Several strains of *B. velezensis* have exhibited the ability to suppress plant pathogens and promote plant growth through various mechanisms such as antimicrobial compound synthesis, phosphate solubilization, nitrogen fixation and phytohormone modulation. For example, the *B. velezensis* LT1 strain displays antifungal activity against the soil-borne fungal plant pathogen *Sclerotium rolfsii* LC1, mediated by the secretion of diverse antimicrobial compounds ( Tang et al. 2024). Similarly, *B. velezensis* strains 5YN8 and DSN012 act as biocontrol agents against *Botrytis cinerea*, a pathogen that causes severe gray mold disease in crops. These strains inhibit fungal spore germination and the growth through the production of certain secondary metabolites and volatile organic compounds ( Jiang et al. 2018). *B. velezensis* HNA3 and SQR9 strains have also been reported to produce several antimicrobial metabolites that help to control plant pathogens ( Zaid et al. 2022; Rabbee et al. 2023). Besides antimicrobial activity, the SQR9 strain implicated in promoting plant growth by enhancing biofilm-mediated root colonization ( Xu et al. 2019). Another strain, *B. velezensis* Ag75, has demonstrated as a biofertilizer and phosphate solubilizer for crops like maize and soybean ( Mosela et al. 2022).

Several studies performed whole genome sequencing to decipher the genetic basis of plants growth promotion and biocontrol capabilities in several *B. velezensis* strains. These studies provide key insights into organism’s capability to produce antimicrobial compounds, enzyme secretion, and secondary metabolites that are involved in plant growth. The genome analysis of *B. velezensis* HNA3 led to establishing several gene clusters associated with promoting plant growth. Among these genes, the major percentage of genes are involved in amino acid metabolism, carbohydrate transport, and secondary metabolite biosynthesis (S. et al. 2022). Similarly, the *B. velezensis* CH1 strain isolated from high-quality oats, exhibited antimicrobial properties contributing to oat growth and resistance to infections. Comparative genomic analysis of CH1 strain revealed 13 gene clusters linked with production of 15 secondary metabolites with antimicrobial properties. Furthermore, the strain harboured numerous putative genes for indole-3-acetic acid (IAA) production, spermidine and polyamine synthesis. These results indicate that the possible applications of *B. velezensis* CH1 as a biofertilizer ( Cheng et al. 2024).

Although multiple *B. velezensis* strains have been reported for plant growth promoting and biocontrol capabilities, most have been isolated from plant-associated environments. Despite these advances, several *B. velezensis* strains remain unexplored, particularly from non-plant environments. In this context, strain *B. velezensis* B26 was isolated from chicken carcass soil, Manipal, Udupi, India (Location: 13.325922, 74.804554). Importantly, this strain demonstrated potent antifungal activity against the opportunistic fungal pathogen *Candida albicans* and the emerging fungal pathogen *Saccharomyces cerevisiae* (Ghurye et al. 2025).

Therefore, the objective of this study is to perform whole genome sequencing and comprehensive genomic analysis of *B. velezensis* B26 to identify genetic traits that contributing to its biotechnological potential.

## Methods

### Whole genome analysis of bacteria

The genome of *B. velezensis* B26 strain was analysed to explore its microbial genomic characteristics. HiMedia Laboratories Pvt Ltd, Maharashtra, INDIA performed the whole-genome analysis.

### Sample preparation workflow

Genomic DNA (gDNA) from *B. velezensis* B26 was isolated using QIAamp DNA Mini Kit (Qiagen). The integrity of the gDNA was assessed spectrophotometrically to measure the A260/280 ratio and its concentration was measured using Qubit broad range dsDNA quantification kit (Thermo Fisher Scientific). For library preparation, 250ng of DNA was processed with the QIASeq FX DNA Kit (Qiagen), following manufacturer’s protocol to generate fragmented, adapter-ligated and indexed libraries. The Illumina NextSeq 550 with 300-cycle paired-end sequencing chemistry was employed for sequencing the finalized library.

### Tapestation analysis of NGS libraries

For fragment analysis, 2 μL of high-sensitivity D1000 sample buffer and 2 μL of the final library sample were mixed using vortexer for 1 min. The mixture was then loaded onto the D1000 ScreenTape and analyzed using the Agilent 4200 TapeStation System. This system employs DNA electrophoresis to separate fragments up to 1000 base pairs. The trace analysis revealed an average fragment size of 301 base pairs, which, along with the library concentration, indicates the library’s suitability for next-generation sequencing.

### Analysis workflow

In data analysis all reads are checked for quality and then the quality control (QC’)-quality reads are passed through the process flows simultaneously. The detailed steps involved in each process flow are included within each section below.

### Reads quality control

Briefly, raw fastq files quality control were verified using FastQc v0.11.9 (Simon Andrews et al. 2010). FastQC report provided individual details on sequence counts, sequence quality histograms, per sequence quality scores, per base sequence content, per sequence GC content, per Base N content, sequence length distribution, sequence duplication levels, overrepresented sequences, adapter content and status checks. The details of individual reports were then added using MultiQC v1.12, to understand the quantity of data obtained for each of the paired read files for an individual sample ( Ewels et al. 2016) (Supplementary file 1). Adapter trimming and quality filtering were performed using fastp tool (v0.12.4) ( Chen et al. 2018). A quality score of Q30 or higher indicates a basecall accuracy of 99.9%, meaning that only 1 in 1000 bases is likely to be incorrect. The resulting files for each read (high quality, adapter-free reads) were used as input for downstream processing.

### Genome assembly and annotations

A denovo assembly using a De-Brujin graph was performed to organize the short DNA reads into longer contiguous sequences, referred to as contigs. These contigs served as a starting material used for performing a genome annotation, facilitating the assignment of functional roles to various genomic regions of the organism under investigation.

The quality of the genome assemblies produced by the Spades ( Bankevich et al. 2012) and Megahit ( Li et al. 2015) assemblers was evaluated using the Quast tool ( Gurevich et al. 2013). Compared with the megahit assembler, the Spades assembler had longer assembled contigs with better N50 values, the Megahit assembly exhibited better genome completeness and compatibility with annotation tools employed in the study. Thus, the Megahit assembly was selected for further downstream analyses.

### RAST Server: Rapid annotations using subsystems technology

The RAST server is an automated platform developed for bacterial genome annotation. It detects protein-coding regions, along with genes coding for ribosomal and transfer RNAs, allots functional roles to these elements ( Aziz et al. 2008). Additionally, it allows comparative analysis through the SEED environment ( Overbeek et al. 2014). The RAST server remains a viable tool for efficient and reliable genome annotation.

Genome annotation was primarily carried out using NCBI Prokaryotic Genome Annotation Pipeline (PGAP), which provides standardized gene prediction and functional characterization. Besides PGAP tool, the RAST server was used to perform subsystem-based annotation. This analysis aids in identification of gene sets grouped under biologically relevant functional categories.

### Pangenome analysis

Genome annotation was carried out using Prokka v1.14.6 (Seemann 2014). It generates standardized GFF3 files, which possess predicted coding sequences (CDS), rRNAs, tRNAs, and functional annotations. Pangenome analysis was subsequently performed using the Roary pipeline v3.13.0 (Page et al. 2015), which aids large-scale analysis of prokaryotic genomes by comparing orthologous genes across different strains. This analysis was carried out using minimum BLASTp identity threshold of 95% for gene clustering. This method facilitates the identification of both core genes (present in ≥99% of genomes), shell genes (present in ≥15% and <95%) and cloud genes (present in <15%), thus provides insights into genome conservation, diversity and evolutionary adaptation.

## Results

### Whole genome analysis of *B. velezensis* B26

We performed a genome analysis of *B. velezensis* B26 to understand the biochemical potential of the organism. Importantly, the assembled genomes may not be 100% complete. However, the assembly generated here meets the standards acceptable to general bioinformatics repositories as part of the data gathered for publication. These whole-genome shotgun data have been provided with the accession number JAYKOV000000000 (NCBI database). The master record data is available from our various Entrez servers. The individual sequences are available from a hyperlink at the bottom of the WGS master record JAYKOV000000000.

The genome assembly method employed was Megahit v. 2023-07-12 tool, which resulted in a complete genome representation with a coverage of 100.0x using Illumina sequencing technology. Genome assemblies were initially produced using both Spades and Megahit, and their quality was evaluated using Quast tool. Although Spades produced longer assembled contigs and higher N50 values, the assembly generated by Megahit exhibited more completeness and was better suited for downstream tools. Therefore, the Megahit assembly was preferred for genome annotation and comparative analysis.

Genome annotation was carried out using the NCBI Prokaryotic Genome Annotation Pipeline (PGAP) ( Haft et al. 2018; Li et al. 2021). The annotation results are summarized in Table 1, which provides an overview of the genome features and information. Table 2 summarizes key assembly statistics, including the number of contigs, total length of the sequenced genome, and the number of annotated proteins.

**Table 1.** Genome annotation data for *Bacillus velezensis* B26.

##Genome-Annotation-Data-START##	
Annotation Provider:	NCBI
Annotation Date:	01/04/2024 08:32:37
Annotation Pipeline:	NCBI Prokaryotic Genome Annotation Pipeline (PGAP)
Annotation Method:	Best-placed reference protein set; GeneMarkS-2+
Annotation Software revision:	6.6
Features Annotated:	Gene; CDS; rRNA; tRNA; ncRNA
Genes (total):	3,891
CDSs (total):	3,835
Genes (coding):	3,776
CDSs (with protein):	3,776
Genes (RNA):	56
rRNAs:	1 (5S)
complete rRNAs:	1 (5S)
tRNAs:	50
ncRNAs:	5
Pseudo Genes (total):	59
CDSs (without protein):	59
Pseudo Genes (ambiguous residues):	0 of 59
Pseudo Genes (frameshifted):	40 of 59
Pseudo Genes (incomplete):	32 of 59
Pseudo Genes (internal stop):	8 of 59
Pseudo Genes (multiple problems):	19 of 59
##Genome-Annotation-Data-END##	
##Genome-Assembly-Data-START##	
Assembly Method:	Megahit v. 2023-07-12
Genome Representation:	Full
Expected Final Version:	Yes
Genome Coverage:	100.0x

**Table 2.** Assembly statistics for *B. velezensis* B26.

# of Contigs:	31
# of Proteins:	3,776
Total length:	3,947,698 bp
BioProject:	PRJNA1060932
BioSample:	SAMN39250459
Keywords:	WGS
Annotation:	Contigs
Organism:	Bacillus velezensis – show lineage
Biosource:	/collected_by = Manipal College of Pharmaceutical Sciences (MCOPS) /collection_date = 2019-12-01 /country = India: Udupi /isolation_source = environmental /mol_type = genomic /strain = B26
WGS:	JAYKOV010000001:JAYKOV010000031
Reference:	Evaluation of bioactivities of the bacterial strain Bacillus velezensis B26: Unpublished
Submission:	Submitted (04-JAN-2024) Pharmaceutical Biotechnology, Manipal College of Pharmaceutical Sciences (MCOPS), MAHE, Madhav Nagar, Manipal, Karnataka 576104, India – Kolathur,K.K.

### Identification of subsystem features by The RAST Server: Rapid Annotations using Subsystems Technology (RAST)

**Figure 1.**
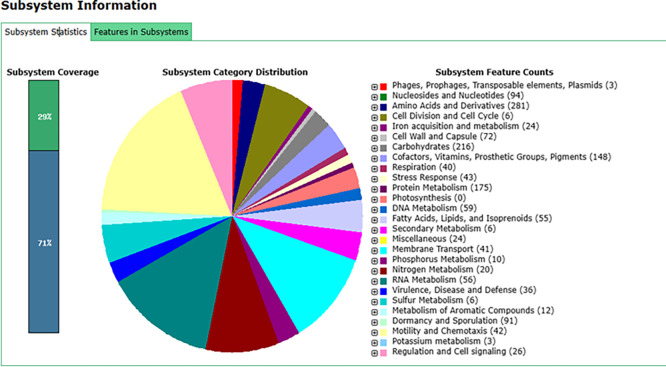
Subsystem statistics of the *B. velezensis* B26 genome annotations. The genome of *B. velezensis* B26 was annotated using the RAST server, and the features in subsystem are compared within the SEED environment. The figure highlights key subsystems identified in the genome, including genes associated with carbohydrate metabolism, stress response, virulence, disease and defense, as well as genes involved in metabolism.

In addition to utilizing the NCBI PGAP, we also submitted the sequenced genome data of *B. velezensis* B26 to the RAST server. This service provides fully automated annotation for bacterial genomes. Using the RAST annotation platform, we recognized genes coding for proteins, ribosomal and transfer RNAs, assigned functional roles to these genes, and predicted the subsystems present in the *B. velezensis* B26 genome (Supplementary Table 1). Furthermore, the annotated genome is presented on a platform that allows for comparative analysis in the SEED environment ( Figure 1).

Our analysis revealed the presence of 216 genes involved in carbohydrate metabolism, 3 genes related to phages, prophages, transposable elements, and plasmids, 43 genes for stress response, and 35 genes linked to virulence, disease and defense. The genes related to the stress response and virulence might be responsible for the biocontrol properties of *B. velezensis* B26 (Supplementary Table 2).

Additionally, several genes involved in metabolic processes were identified, including 59 genes related to DNA metabolism, 10 related to phosphorus metabolism, 20 related to nitrogen metabolism, 6 related to sulfur metabolism, 12 related to metabolism of aromatic compounds, 3 related to potassium metabolism, 10 related to phosphorus metabolism, and 24 related to acquisition and metabolism. These metabolic pathways suggest potential biofertilizer activity (Supplementary Table 2).

### Functional implications of subsystem genes

Subsystem-based genome annotation of *B. velezensis* B26 revealed gene sets that may contribute to its biocontrol and biofertilizer activities. The genome contains 43 genes related to stress response and 35 genes involved in virulence, disease, and defense, suggesting the strains ability to tolerate environmental stress and inhibit plant pathogens. Additionally, the presence of genes involved in phosphorus (10 genes), nitrogen (20 genes), and sulfur metabolism (6 genes) indicates the stains potential for nutrient acquisition and its role as a biofertilizer. Altogether, these gene sets provide valuable insights into the biotechnological potential of *B. velezensis* B26.

### *B. velezensis* B26 pangenome analysis

Pangenome analysis provides valuable understandings into the genome of prokaryotes. Using Roary software, a large-scale pangenomes are constructed by identifying both core and accessory genes. This method aids in understanding the conserved genes within an organism, as well as accessory genome, to understand the fundamentals linked with natural selection and evolutionary dynamics.

**Figure 2.**
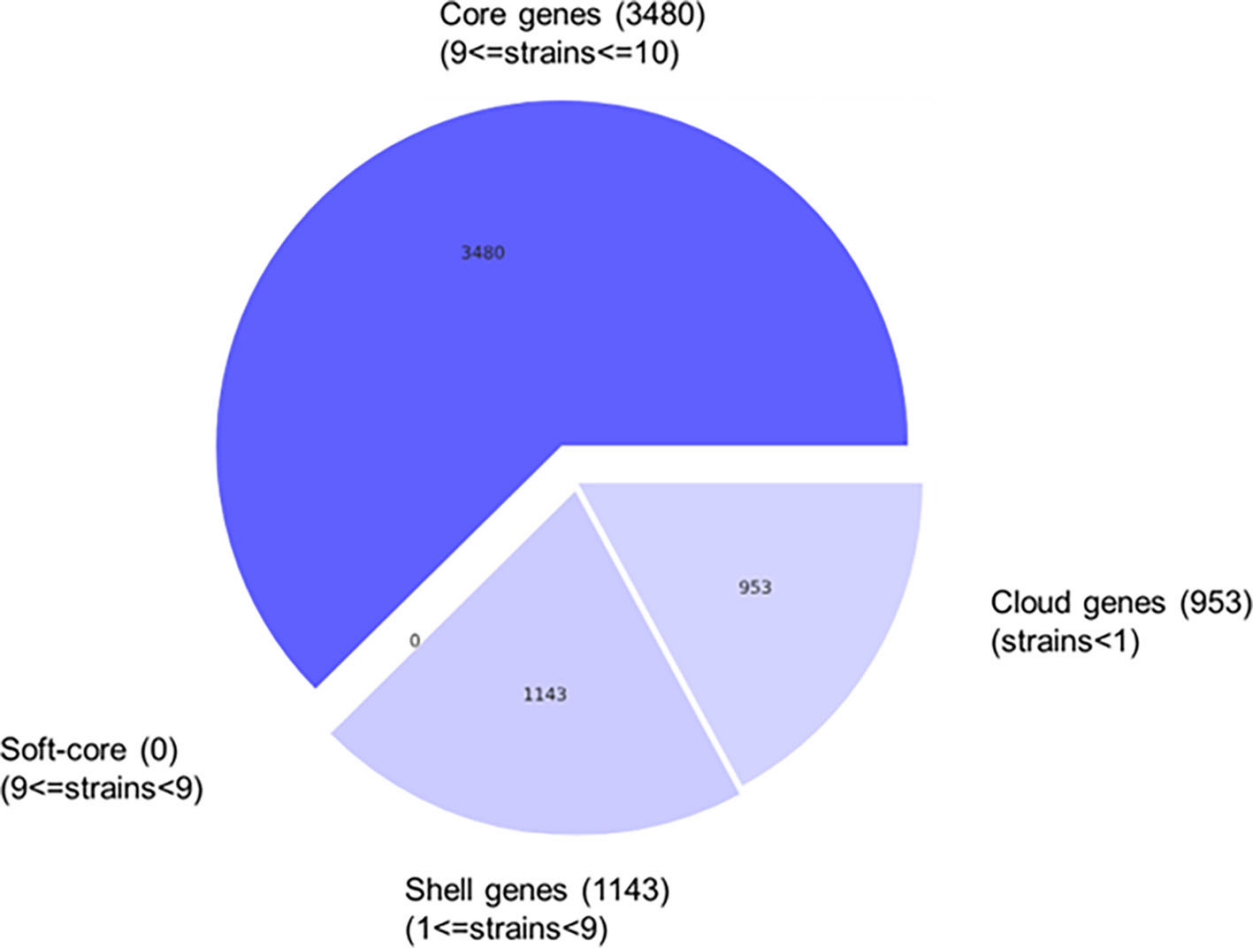
Pie chart represents group of genes. The pie chart represents groups of genes that were classified into various groups based on their prevalence in the pangenome analyzed.

Pangenome analysis of *B. velezensis* B26 identified a total of 5,576 genes. Among these genes, 3,480 genes were considered core genes, present in 99% to 100% of strains. Additionally, 1,143 genes were classified as shell genes, which were present in 15% to 95% of strains, while 953 genes were identified as cloud genes, found in fewer than 15% of the analyzed strains ( Figure 2, Table 3). These results indicate that a major portion of the *B. velezensis* B26 genome is conserved, while significant proportion of cloud genes (953 out of 5,576), may represent strain-specific or rare genes. Although many of the genes remains uncharacterized, these accessory genes might contribute to the distinctive biocontrol and plant growth promoting capabilities of *B. velezensis* B26.

**Table 3.** Pangenome analysis of *B. velezensis* B26.

	Gene_type	Criteria	Number_of_genes
1	Core genes	(99% <= strains <= 100%)	3480
2	Soft core genes	(95% <= strains < 99%)	0
3	Shell genes	(15% <= strains < 95%)	1143
4	Cloud genes	(0% <= strains < 15%)	953
5	Total genes	(0% <= strains <= 100%)	5576

## Conclusion

The whole-genome analysis, genome annotation, and pangenome analysis of *B. velezensis* B26 deciphered the key genes responsible for its biotechnological applications. A huge number of protein coding genes (3776) responsible for several cellular functions were identified. Importantly, the presence of gene cluster coding for stress response, virulence, and defense indicates that *B. velezensis* B26 can effectively inhibit plant pathogens. Further, the presence of genes associated with nitrogen, phosphorus, and sulfur metabolism might suggest the strain’s ability to act as a biofertilizer, supporting plant growth. Altogether, these findings can pave the way for further exploration of *B. velezensis* B26 in agricultural applications.

## Ethics and consent

Ethical approval and consent were not required.

## Data Availability

NCBI Nucleotide Database: Whole-genome sequencing data of Bacillus velezensis B26. Accession number JAYKOV000000000; https://www.ncbi.nlm.nih.gov/nuccore/JAYKOV000000000. Contigs view; https://www.ncbi.nlm.nih.gov/Traces/wgs/JAYKOV01?display=contigs. Whole-genome shotgun project; https://www.ncbi.nlm.nih.gov/Traces/wgs/JAYKOV01. These data support the findings of this study and are publicly accessible. The complete genome sequence and associated supplementary data for *Bacillus velezensis* B26 are available on **Figshare**. **Figshare**: Supplementary file 1 for the complete genome sequences of *Bacillus velezensis* B26 study. DOI: 10.6084/m9.figshare.28107950. The project contains the following underlying data:
•
**Supplementary Table 1**: Complete genome sequences of *Bacillus velezensis* B26. DOI: 10.6084/m9.figshare.28107941. •
**Supplementary Table 2**: Complete genome sequence for *Bacillus velezensis* B26. DOI: 10.6084/m9.figshare.28107947. Data are available under the terms of the Creative Commons Attribution 4.0 International license (CC-BY 4.0).
