# Supplementary material for: The complete genome sequences of Bacillus velezensis B26: a promising biocontrol agent and biofertilizer
Source: F1000Res. 2025 Jul 8;14:170. Originally published 2025 Feb 6. [Version 2] doi: 10.12688/f1000research.160546.2 (PMC12149806; doi:10.12688/f1000research.160546.2)
Supplement: Peer review history

# Reviewer report: Reviewer response for version 2

**DOI:** 10.5256/f1000research.184085.r397488

**Published:** 2025-08-23

**Authors:** Surajit Basak

**Affiliations:** 1 ICMR - National Institute for Research in Bacterial Infections, Beliaghata Kolkata, India

The revised manuscript may be accepted for indexing.

Are the datasets clearly presented in a usable and accessible format, and the assembly and annotation available in an appropriate subject-specific repository?

Yes

Are sufficient details of the sequencing and extraction, software used, and materials provided to allow replication by others?

No

Are the rationale for sequencing the genome and the species significance clearly described?

Yes

Are the protocols appropriate and is the work technically sound?

Partly

Reviewer Expertise:

NA

I confirm that I have read this submission and believe that I have an appropriate level of expertise to confirm that it is of an acceptable scientific standard.

# Reviewer report: Reviewer response for version 2

**DOI:** 10.5256/f1000research.184085.r397487

**Published:** 2025-08-23

**Authors:** Diego Sauka

**Affiliations:** 1 Instituto Nacional de Tecnología Agropecuaria (INTA), Hurlingham, Argentina

The authors have successfully addressed all the requests and comments made by this reviewer.

Are the datasets clearly presented in a usable and accessible format, and the assembly and annotation available in an appropriate subject-specific repository?

Yes

Are sufficient details of the sequencing and extraction, software used, and materials provided to allow replication by others?

Yes

Are the rationale for sequencing the genome and the species significance clearly described?

Yes

Are the protocols appropriate and is the work technically sound?

Yes

Reviewer Expertise:

Microbiology; genomics; biological control of pests

I confirm that I have read this submission and believe that I have an appropriate level of expertise to confirm that it is of an acceptable scientific standard.

# Reviewer report: Reviewer response for version 1

**DOI:** 10.5256/f1000research.176457.r384406

**Published:** 2025-06-09

**Authors:** Diego Sauka

**Affiliations:** 1 Instituto Nacional de Tecnología Agropecuaria (INTA), Hurlingham, Argentina

The article provides valuable insights into *Bacillus velezensis* B26, a bacterium with demonstrated capabilities in pathogen control and plant growth promotion. However, the introduction requires greater clarity and focus. Although the authors include numerous examples of *B. velezensis* strains with antimicrobial and growth-promoting properties, they fail to effectively connect these examples to the study’s relevance. Moreover, the broader purpose and scientific significance of the work within the context of previous research remain unclear.

 The text contains redundancies, such as repeated mentions of the bacterium's ability to produce secondary metabolites, without offering a nuanced or differentiated discussion. Furthermore, some references are poorly integrated, as they are merely listed without elaborating on how they support the article’s hypotheses. The transitions between antimicrobial properties, plant growth promotion, and genomic studies are also disjointed, making the narrative difficult to follow.

 The introduction would benefit from a clearly stated hypothesis or specific objective emphasizing the biotechnological relevance of *B. velezensis* B26. Establishing a stronger connection between the genomic findings and their practical applications would significantly enhance the cohesion of the text.

**Specific comments**

**Materials & Methods (M&M)**

 The authors state that they used two different assemblers for genome assembly. However, the results of the SPAdes assembly are not shown in the Results section. This should be clarified. Additionally, the authors include a paragraph explaining the RAST Server but fail to specify its purpose in the study. Later, in the Results section, they mention using the Prokaryotic Genome Annotation Pipeline (PGAP). This inconsistency requires clarification.

**Results section**

 The number of tables and figures could be reduced. For example:

- Figure 1 is unnecessary as it repeats information already described in the M&M section.
- Tables 1 and 2 could be combined for better conciseness.
- Figure 2 is not particularly informative, as it presents general information from the RAST Server without any meaningful analysis.

A better presentation focusing specifically on genes associated with pathogen control and plant growth promotion would improve the article significantly. Highlighting these capabilities with well-analyzed data would make the study more impactful and engaging.

Are the datasets clearly presented in a usable and accessible format, and the assembly and annotation available in an appropriate subject-specific repository?

Yes

Are sufficient details of the sequencing and extraction, software used, and materials provided to allow replication by others?

Yes

Are the rationale for sequencing the genome and the species significance clearly described?

Yes

Are the protocols appropriate and is the work technically sound?

Yes

Reviewer Expertise:

Microbiology; genomics; biological control of pests

I confirm that I have read this submission and believe that I have an appropriate level of expertise to confirm that it is of an acceptable scientific standard, however I have significant reservations, as outlined above.

# Reviewer report: Reviewer response for version 1

**DOI:** 10.5256/f1000research.176457.r384408

**Published:** 2025-06-09

**Authors:** Surajit Basak

**Affiliations:** 1 ICMR - National Institute for Research in Bacterial Infections, Beliaghata Kolkata, India

The study delivers an organized, useful genome report on Bacillus velezensis B26 as it narrates about the possible use as a biocontrol agent and biofertilizer.

 The use of genome assembly and annotation pipeline pipelines and pangenome analysis provide more insights into the genome sequences.

 There are some issues that will help to improve the depth of the manuscript.

 1) Add a brief in the introduction by stating importance of giving more emphasis on B26 compared to other Bacillus velezensis strains.

 2) Elaborate the methodology section with detailed description of the work done and the tools used for performing them along with proper citations of the tools.

 3) No methodology is given for pangenome analysis, give a brief methodology of pangenome analysis with proper citations.

 4) Why two assembly pipelines have been used is not clear. Also, there are disparity of information provided in the methodology section “The quality of the genome assemblies produced by the Spades (Bankevich et al. 2012) and Megahit (Li et al. 2015) assemblers was evaluated using the Quast tool (Gurevich et al. 2013).

 Compared with the megahit assembler, the Spades assembler had longer assembled contigs with better N50 values.

 Hence, the assembly generated by Spades was selected for further downstream analyses.” and in the results section “The genome assembly method employed was Megahit v. 2023-07-12 tool, the genome representation was full….” - Explain this.

 5) Subsystem information is given. Discuss about the gene or gene set responsible for B. velezensis B26.

 6) Along with the table of pangenome analysis add plots showing the gene distribution. Is there any unique feature of the Bacillus velezensis B26 strain?

Are the datasets clearly presented in a usable and accessible format, and the assembly and annotation available in an appropriate subject-specific repository?

Yes

Are sufficient details of the sequencing and extraction, software used, and materials provided to allow replication by others?

No

Are the rationale for sequencing the genome and the species significance clearly described?

Yes

Are the protocols appropriate and is the work technically sound?

Partly

Reviewer Expertise:

NA

I confirm that I have read this submission and believe that I have an appropriate level of expertise to confirm that it is of an acceptable scientific standard, however I have significant reservations, as outlined above.

# Response

**Published:** 2025-06-19

**Authors:** KIRAN KOLATHUR

**Affiliations:** Pharmaceutical Biotechnology, Manipal Academy of Higher Education, Manipal, Karnataka, India

**Point-by-point response to Comments**

 We have highlighted all the changes made in the manuscript.

 We thank the reviewers for the comments and tried to present the data in a detailed manner and the points raised are discussed below.

***Reviewer 2:***

*The study delivers an organized, useful genome report on Bacillus velezensis B26 as it narrates about the possible use as a biocontrol agent and biofertilizer. The use of genome assembly and annotation pipeline pipelines and pangenome analysis provide more insights into the genome sequences.*

*Response: *We thank the reviewer for the comments

*There are some issues that will help to improve the depth of the manuscript.*

*1) Add a brief in the introduction by stating the importance of giving more emphasis on B26 compared to other Bacillus velezensis strains.*

**

 Response 1: As mentioned, we have revised the introduction to highlight why strain B26 was chosen for genome sequencing. Specifically, we emphasize its unique origin (chicken carcass soil), its antifungal spectrum that includes both agricultural and human pathogens, and its potential for broader biotechnological applications. These features distinguish B26 from the most previously characterized B. velezensis strains and justify its genomic investigation. We have modified the introduction accordingly.

*2) Elaborate the methodology section with detailed description of the work done and the tools used for performing them along with proper citations of the tools.*

 Response 2: We thank the reviewer for the comment; we have added detailed description to the methodology (Page 3 & 4).

*3) No methodology is given for pangenome analysis, give a brief methodology of pangenome analysis with proper citations.*

 Response 3: We thank the reviewer for the comment; we have added detailed description to the methodology (Page 4).

*Pangenome Analysis*

*“Genome annotation was carried out using Prokka v1.14.6 (Seemann 2014). It generates standardized GFF3 files, which possess predicted coding sequences (CDS), rRNAs, tRNAs, and functional annotations. Pangenome analysis was subsequently performed using the Roary pipeline v3.13.0 (Page et al. 2015), which aids large-scale analysis of prokaryotic genomes by comparing orthologous genes across different strains. This analysis was carried out using minimum BLASTp identity threshold of 95% for gene clustering. This method facilitates the identification of both core genes (present in ≥99% of genomes), shell genes (present in ≥15% and <95%) and cloud genes (present in <15%), thus provides insights into genome conservation, diversity and evolutionary adaptation.”*

*4) Why two assembly pipelines have been used is not clear. Also, there are disparity of information provided in the methodology section “The quality of the genome assemblies produced by the Spades (Bankevich et al. 2012) and Megahit (Li et al. 2015) assemblers was evaluated using the Quast tool (Gurevich et al. 2013). Compared with the megahit assembler, the Spades assembler had longer assembled contigs with better N50 values. Hence, the assembly generated by Spades was selected for further downstream analyses.” and in the results section “The genome assembly method employed was Megahit v. 2023-07-12 tool, the genome representation was full….” - Explain this.*

 Response 4: We thank the reviewer for pointing out the discrepancy in the methods section (Page no.4) and results section (Page no.5). We have modified as below

*“The genome assembly method employed was Megahit v. 2023-07-12 tool, which resulted in a complete genome representation with a coverage of 100.0x using Illumina sequencing technology. Genome assemblies were initially produced using both Spades and Megahit, and their quality was evaluated using Quast tool. Although Spades produced longer assembled contigs and higher N50 values, the assembly generated by Megahit exhibited more completeness and was better suited for downstream tools. Therefore, the Megahit assembly was preferred for genome annotation and comparative analysis”.*

*5) Subsystem information is given. Discuss the gene or gene set responsible for B. velezensis B26.*

 Response 5: We thank the reviewer for the comment; we have added detailed as below (Results section, page no 6)

*Functional implications of subsystem genes*

*“Subsystem-based genome annotation of B. velezensis B26 revealed gene sets that may contribute to its biocontrol and biofertilizer activities. The genome contains 43 genes related to stress response and 35 genes involved in virulence, disease, and defense, suggesting the strain’s ability to tolerate environmental stress and inhibit plant pathogens. Additionally, the presence of genes involved in phosphorus (10 genes), nitrogen (20 genes), and sulfur metabolism (6 genes) indicates the stain’s potential for nutrient acquisition and its tole as a biofertilizer. Altogether, these gene sets provide valuable insights into the biotechnological potential of B. velezensis B26”.*

*6) Along with the table of pangenome analysis add plots showing the gene distribution. Is there any unique feature of the Bacillus velezensis B26 strain?*

 Response 6: We thank the reviewer for this helpful suggestion. In response, we have now included additional visualizations to support the pangenome analysis:

*A pie chart (now added as Figure 2) illustrates the distribution of gene categories across the pangenome, including core, shell, cloud, and soft-core genes.*

*“Pangenome analysis of B. velezensis B26 identified a total of 5,576 genes. Among these genes, 3,480 genes were considered core genes, present in 99% to 100% of strains. Additionally,1, 143 genes were classified as shell genes, which were present in 15% to 95% of strains, while 953 genes were identified as cloud genes, found in fewer than 15% of the analysed strains (Figure 2, Table 3). These results indicate that a major portion of the B. velezensis B26 genome is conserved, while significant proportion of cloud genes (953 out of 5,576), may represent strain-specific or rare genes. Although many of the genes remains uncharacterized, these accessory genes might contribute to the distinctive biocontrol and plant growth promoting capabilities of B. velezensis B26”.*

# Reviewer report: Reviewer response for version 1

**DOI:** 10.5256/f1000research.176457.r365574

**Published:** 2025-03-31

**Authors:** Saranya Nallusamy

**Affiliations:** 1 Tamil Nadu Agricultural University, Coimbatore, Tamil nadu, India

The manuscript titled "The complete genome sequences of *Bacillus velezensis* B26: a promising biocontrol agent and biofertilizer" presents the whole-genome sequencing of *B. velezensis* B26, a strain isolated from chicken carcass soil in Udupi, India. The study highlights the strain’s biocontrol potential and plant growth-promoting abilities, emphasizing its antifungal properties and enzymatic activities. Whole-genome sequencing was performed using Illumina technology, and genome annotation identified multiple functional genes linked to metabolic processes, stress response, and biocontrol properties. 

*Bacillus velezensis* has multiple applications in agriculture, biotechnology, and medicine.  It plays a key role in plant growth promotion, biocontrol activity, and industrial applications.  Its genome sequencing helps in understanding its potential for various applications, particularly in sustainable agriculture.

             This article reports the genome sequence of strain B26 to explore its potential in agriculture and biocontrol.  Comparative genomics with other strains highlights unique genetic features that contribute to its biocontrol capabilities. Pan genome analysis reported the core and shell genes which is crucial for multiple applications in microbiology, biotechnology, and evolutionary studies.

             The study employed appropriate and standard methods like SPAdes for genome assembly and RAST & NCBI PGAP for genome annotation. The genome sequence and annotation data have been deposited in NCBI under accession number JAYKOV000000000.

 Genome sequencing highlights its potential for secondary metabolite production, making it a strong candidate for synthetic biology and metabolic engineering.

Are the datasets clearly presented in a usable and accessible format, and the assembly and annotation available in an appropriate subject-specific repository?

Yes

Are sufficient details of the sequencing and extraction, software used, and materials provided to allow replication by others?

Yes

Are the rationale for sequencing the genome and the species significance clearly described?

Yes

Are the protocols appropriate and is the work technically sound?

Yes

Reviewer Expertise:

Plant genomics and transcriptomics, structural bioinformatics

I confirm that I have read this submission and believe that I have an appropriate level of expertise to confirm that it is of an acceptable scientific standard.
